# HealthPass: a contactless check-in and adaptive access control system for lowering cluster infection risk in public health crisis

**DOI:** 10.3389/fpubh.2024.1448901

**Published:** 2024-12-12

**Authors:** Guofeng Luo, Yufei Wang, Linghong Hong, Xin He, Jiaru Wang, Qu Shen, Cheng Wang, Longbiao Chen

**Affiliations:** ^1^Fujian Key Laboratory of Sensing and Computing for Smart Cities, School of Informatics, Xiamen University, Xiamen, China; ^2^Department of Drug Clinical Trial Institution, Xiang'an Hospital of Xiamen University, School of Medicine, Xiamen University, Xiamen, China; ^3^Department of Nursing, Xiamen University, Xiamen, China

**Keywords:** contactless check-in, mobile application, location-based services, social distance, public health

## Abstract

**Introduction:**

Ensuring effective measures against the spread of the virus is paramount for educational institutions and workplaces as they resume operations amidst the ongoing public health crisis. A touchless and privacy-conscious check-in procedure for visitor assessment is critical to safeguarding venues against potential virus transmission.

**Methods:**

In our study, we developed an interaction-free entry system featuring anonymous visitors who voluntarily provide data. This system introduces an adaptable venue entry management mechanism that accounts for both visitors' potential risk and the venue's capacity, aiming to curb the risk of localized infections. We assess visitors' liability based on their voluntarily provided data through radar map analysis. Additionally, we evaluate the venue's situation by quantifying its risk from multiple dimensions. A queuing model is then employed to control visitor access adaptively based on visitors' liability and the venue's availability.

**Results:**

Since May, our university campus has been the operational site for the implemented system, catering to the needs of visitors across distinct venues. Using real-world implementation, we conduct a series of simulation experiments and case studies to verify the effectiveness of the HealthPass system in lowering infection risks.

**Discussion:**

The system has demonstrated its capacity to reduce infection risks by adapting visitor entry procedures based on individual risk factors and venue conditions. Our results suggest that the integration of a dynamic queuing model and real-time data analysis can effectively manage the flow of visitors while ensuring public health safety.

## 1 Introduction

The outbreak of public health incidents can cause a global crisis, such as the SARS epidemic in 2002, the H1N1 influenza pandemic in 2009, and COVID-19 in 2019. As of April 29, 2021, COVID-19 infections had been reported in more than 210 countries and regions worldwide, exceeding 150.57 million cases, with a death toll of over 3,167,343^1^. Although the epidemic has passed, we still need to be prepared for potential threats and we need effective ways to control the spread of public health diseases. This involves several aspects, such as regulating the source of infection, eradicating transmission routes, and safeguarding susceptible individuals. The findings of extensive investigations suggest that implementing crowd density reduction measures in specific locations can effectively reduce the risk of cluster infections (1). However, finding a suitable equilibrium between implementing preventative crowd management measures and restoring the normal functioning of essential civic and commercial activities remains challenging. As an illustration, following the resumption of economic activities, Beijing witnessed a cluster infection outbreak at the Xinfadi Wholesale Market June in 2020 (2). Given that a public health crisis could last for months or even years (3), we must explore practical methods of regulating entry to venues providing essential services and recreational activities, aiming to minimize the risk of cluster infections when ensuring public accessibility.

Numerous strategies have been suggested globally for venue access management, with several gaining prevalence nowadays. The most conventional approach involves visitors using paper and pen to register their entry into a location. This could potentially result in a cluster infection as a consequence of shared contact with the sign-in materials. Furthermore, recording and processing the data from the sign-in sheet can be time-consuming, rendering it challenging to enforce immediate access control. An increasingly favored substitute is the display of health codes for venue entry (4). However, the shortcoming of this approach is its inability to maintain a record of individuals entering the premises, which may result in a lack of headcount data. Another method, utilized by certain schools and hotels, involves visitors recording their check-in details via an access control device using ID cards (5). This may add to the responsibilities of venue managers, as they are tasked with installing or temporarily adjusting a piece of similar equipment in advance, resulting in increased maintenance expenses, and the unique data they generate cannot be exchanged with other establishments.

**Figure 1:**
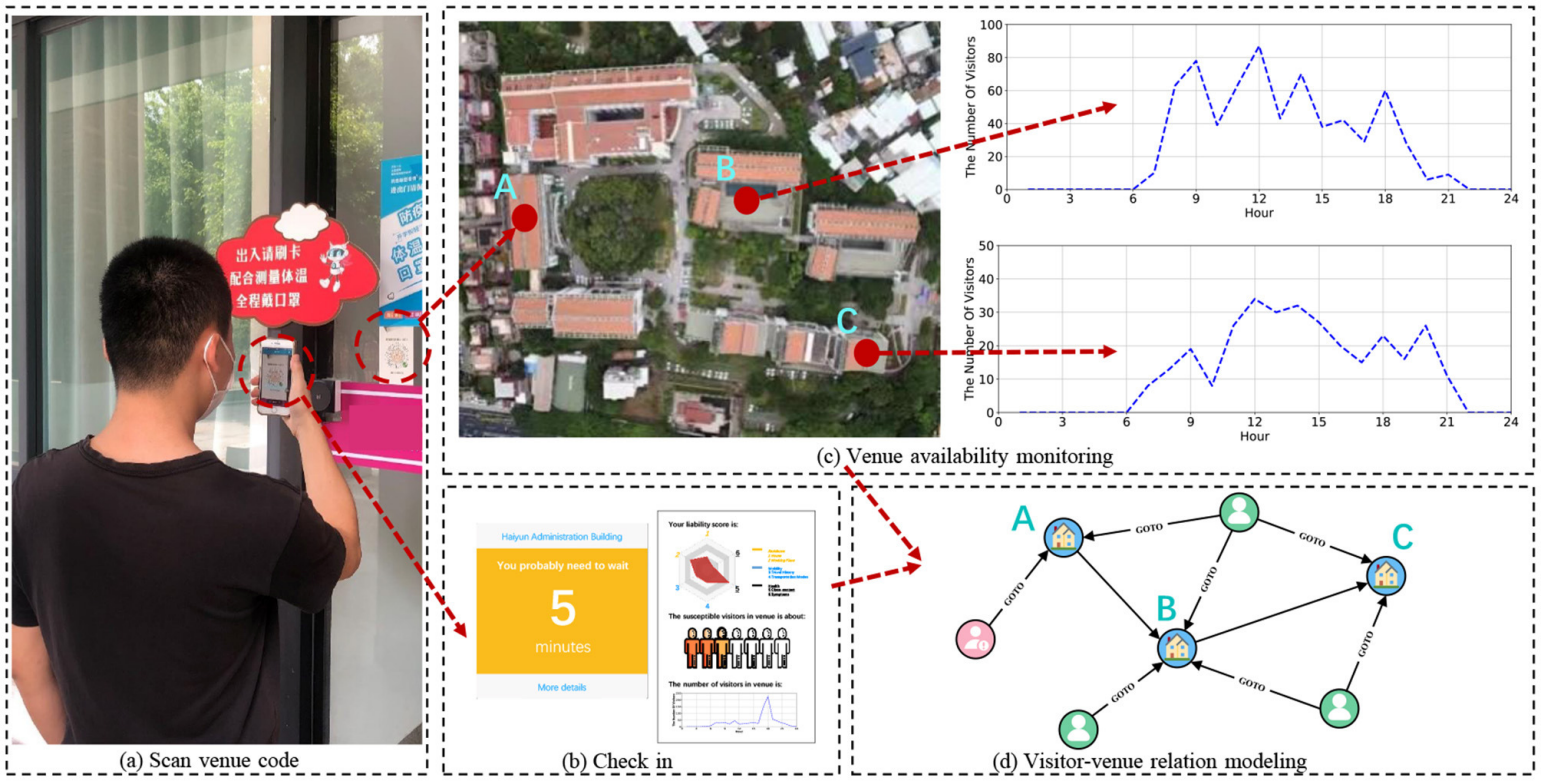
The implementation of the HealthPass system in real-world scenarios. **(a)** Upon arrival, visitors have the option to swiftly scan the QR code using their mobile phone camera to finalize and submit a check-in request. Through this procedure, the venue can assess the liability of visitors derived from the information they furnish. **(b)** The HealthPass system provides real-time updates on venue status and the liability of visitors' profiles. According to this information, the system dynamically manages visitor entry, granting immediate entrance or placing them on a waiting list. **(c, d)** The registration information gets stored in a graph database.

To achieve contactless check-in and efficient access control, while overcoming the limitations of the aforementioned approaches, we devise and execute a system named *HealthPass*. Figure 1 depicts the process of creating and displaying an entry QR code that contains the unique identifier of the venue. Upon arrival, visitors have the option to swiftly scan the QR code using their mobile phone camera to fulfill and send a check-in request. Through this procedure, the venue can evaluate the liability of visitors by considering the data they willingly provide (Figure 1a). The system employs data regarding the current venue occupancy status and the visitor's liability information (Figure 1b) to dynamically regulate venue access, either providing immediate entrance or placing them on a waiting list (Figure 1c). While developing the HealthPass system, our goal is to tackle the subsequent hurdles:

1. **How to profile visitor's liability?** During a public health emergency, visitor-related liability data can be multifaceted and extensive, encompassing aspects such as their residence, places visited, and presence of specific symptoms, among others. However, gathering excessive details might infringe on visitors' privacy rights and elongate the check-in procedure. Additionally, formulating an all-encompassing approach to determine visitors' liability, considering the provided data poses a considerable challenge.
2. **How to estimate the venue's availability?** Amidst a public health catastrophe, the accessibility of a venue can be influenced by various elements, like its size and congestion level. Furthermore, the presence of patrons within the venue and the surrounding neighborhood also impact its accessibility. However, gathering and quantifying this kind of data poses challenges. As a result, there is a need for an efficient model that can evaluate venue accessibility from diverse perspectives.
3. **How to optimize venue access control strategy?** To prevent a surge of individuals entering the venue during high-traffic periods, it's necessary to allocate appropriate entry times based on various conditions. Given the dynamic nature of visitor liability and venue availability, devising an optimal strategy for venue access control presents a significant challenge.

Considering the previously discussed concerns and aims, this paper primarily contributes in the following ways:

- We delve into the issue of dynamic venue access management during public health crises, facilitating contactless check-in and reducing cluster infection risks in an efficient, cost-effective, and automated manner.
- We develop and introduce the HealthPass system. Initially, we implement QR codes to expedite venue check-ins and store the relevant data in a graph database. Second, we employ a radar-based approach to assess visitors' liability and combine their risk with venue connectivity and crowdedness levels to gauge venue availability. To mitigate congestion, we suggest a priority queue-based approach that considers both the liability of visitors and the availability of venues for access control.
- We implement the HealthPass system within an actual university campus, amassing abundant anonymous usage data for analysis, commencing from its launch in May 2020. Drawing from the accumulated data, we carry out a series of simulation experiments to evaluate the effectiveness of our system in lowering infection risks. In addition, we conduct a system usability scale survey (SUS) to prove that our system has good usability and perform two case studies on the campus to assess the performance of our system.

## 2 Related work

A multitude of infection-prevention applications have surfaced due to the COVID-19 outbreak. Traditional access control methods are usually rules-based. As an illustration, Zhang et al. (6) introduced a symptom screening application that empowers employees to conduct self-screening for COVID-19 symptoms prior to entering the facility. García et al. (7) present a simple and versatile method for calculating the maximum capacity of public spaces constrained to social distancing. Drew et al. (8) introduced a mobile app designed to collect data from individuals with symptoms and track the disease's progression through daily voluntarily providing health data.

Furthermore, numerous contact-tracing applications utilizing mobile location technology have surfaced. As an instance, the Singaporean authorities launched the TraceTogether app (9), which employs the Bluetooth functionality of smartphones to retain data regarding the proximity of other smartphones to your device. These accumulated data aid the government in conducting *contact tracing* procedures, helping to trace individuals who may have had possible exposure to the virus. China's Ali-pay released the Health Code app (4, 10), which generates a unique digital QR code for each user, taking into account their health status, travel records, and potential interactions with individuals from affected regions.

Access control systems play a crucial role in various applications, particularly in preventing the spread of viruses and managing personnel movement. Depending on the method of authentication, access control systems can be classified into biometric and non-biometric systems.

Biometric technology has been widely adopted in access control systems, especially in high-security environments and during public health crises. By utilizing technologies such as fingerprint, iris, or facial recognition, biometric systems provide highly reliable methods of identity verification. Research indicates that biometric systems have significant advantages in reducing human intervention and enhancing security. The typical process flow of a biometric system includes data acquisition, feature extraction, matching and verification, and access control decision-making. Initially, sensors acquire the user's biometric data during the data acquisition phase; then, feature extraction converts this data into feature vectors suitable for matching. Subsequently, the system compares these features with pre-registered data in the database. If a match is successful, the user is granted access. Although biometric systems offer a high level of security, they also face challenges related to privacy protection, data processing, and technical costs.

Non-biometric systems primarily rely on the user's knowledge (such as passwords), possessions (such as smart cards or keys), or behavioral characteristics (such as typing rhythm) to verify identity. The advantages of such systems include simplicity of implementation, lower costs, and widespread use in many traditional scenarios. The typical process flow of non-biometric systems includes inputting authentication information, verification, and access control decision-making. Users prove their identity by entering a password or using a smart card, and the system compares the input data with pre-registered data. If the match is successful, access is granted. However, these systems may be vulnerable to attacks, such as credential theft or loss, and therefore have certain limitations in high-security environments.

Moreover, some use machine learning-based approaches to control human flow (11, 12). For instance, Durań-Polanco and Siller (13) introduced an approach using a point-of-interest (POI) recommendation system, which offers nearby safe alternatives when a user requests to visit a specific POI. This system aims to monitor and regulate crowd levels in indoor locations or POI. The work by Punn et al. (14) suggests a deep learning-driven framework that automates the process of surveilling social distancing by analyzing surveillance video.

In recent years, Rahman et al. (15) explored the application of contactless services in the hospitality industry, particularly in the post-COVID-19 era, highlighting the potential of these technologies to guide future research directions. The study emphasized the importance of contactless technology and its potential to provide a safe and seamless user experience. Meanwhile, Kaur et al. (16) reviewed various forms of access control systems and their feasibility in the context of COVID-19. This comprehensive review covered both biometric and non-biometric systems, including technologies such as fingerprint, facial, palm print, vein pattern, iris, and retinal recognition. The study also analyzed the advantages and limitations of these technologies, providing a solid theoretical foundation for this research.

We also clarify the differences between these works and the proposed work using the Table 1.

**Table 1:** Conpetitive product analysis.

**Method**	**User profiling**	**Dynamic access control**	**Venue availability**	**Privacy**
MVP (6)	Voluntarily providing	Rule-based	N/A	N/A
Health code (4, 10)	State of health,travel history, contact record	Rule-based	N/A	N/A
Estimate capacity (7)	N/A	N/A	Maximum capacity	N/A
ABM (13)	Voluntarily providing	Recommendation system	Data-driven	N/A
Monitor distancing (14)	N/A	Monitor distance	N/A	N/A
HealthPass (ours)	Voluntarily providing	Heuristic	Data-driven	Anonymous collection

MVP: a digital symptom screening and attestation application that would enable employees who work onsite at a facility that provides direct patient care to be able to self-screen for symptoms concerning COVID-19 infection prior to being allowed into the facility (6).

Health Code: the Health Code App assigns a digital QR code to each user according to their state of health, travel history, and whether they contacted people from epidemic areas (4, 10).

Estimate Capacity: a simple and versatile method for calculating the maximum capacity of public spaces constrained to social distancing, following the recommended measures of the WHO due to the COVID-19 pandemic outbreak (7).

ABM: based on a POI recommendation system that suggests the nearest safe options upon request of a particular POI to visit by the user to monitor and manage crowd levels in interior places or point-of-interests (POI) (13).

Monitor Distancing: a deep learning-based framework for automating the task of monitoring social distancing using surveillance video (14).

## 3 Preliminaries and framework

### 3.1 Preliminaries

#### 3.1.1 Visitor liability

We assess the liability score of each visitor to a venue from the following three aspects, i.e., *whether they live in high-risk regions, whether they have traveled to high-risk regions*, and *whether they have specific symptoms or close contact to confirmed cases*. When the visitor's risk is higher, their corresponding liability will be lower. We profile visitors' liability based on their voluntarily providing sheets to identify the source of infection.

#### 3.1.2 Venue availability

We define the venue availability as a safety metric of the venue which is the probability of a random visitor not getting infected in random contact with other visitors in the venue. We estimate the venue availability metric to control the route of transmission according to three factors, i.e., the in-venue visitor's risk the density of visitors in the venue, the venue's connectivity the connection intensity with neighboring high-risk venues, and the venue's crowdedness the number of low-liability visitors in the venue.

#### 3.1.3 Access control

We define access control as the method which can grant different priorities to different visitors to protect susceptible populations in the venue. The system takes both visitor liability and venue availability into consideration and arranges an appropriate time for visitors to enter the venue.

By adhering to these three principles, the HealthPass system ensures epidemic infection prevention and reduces the risk of cluster infections within venues.

### 3.2 Framework

**Figure 2:**
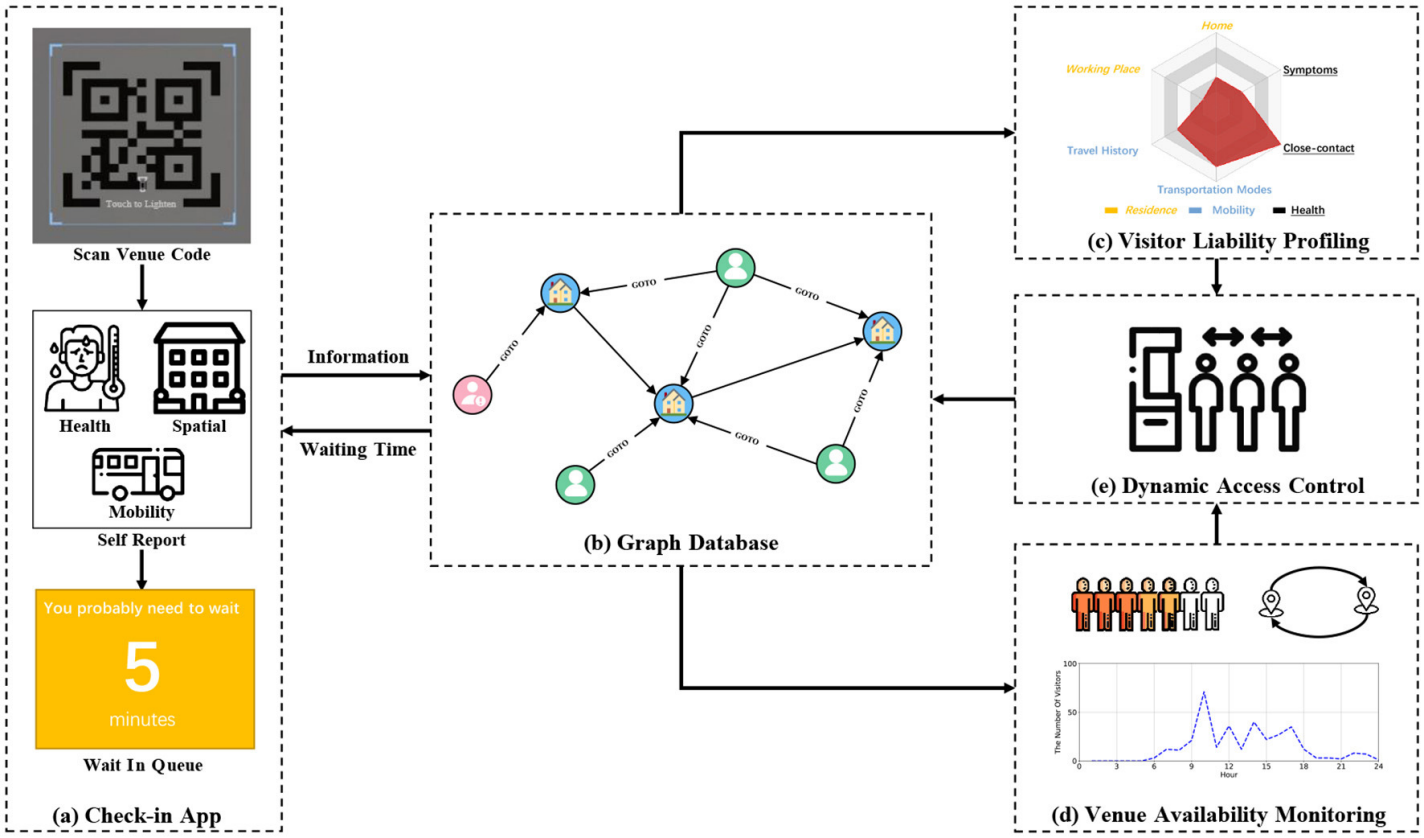
Framework of the HealthPass system. **(a)** The visitor scans the QR code and completes the voluntarily providing forms. **(b)** The data is stored in a graph database. **(c)** The application employs the radar map to assess visitor liability. **(d)** The system gauges the venue availability. **(e)** The system dynamically controls the visitor's access by granting them access.

Illustrated in Figure 2, we introduce HealthPass, a novel solution enabling contactless check-in and dynamic access control. To initiate the process, the visitor utilizes their mobile device to scan the venue's QR code, completes self-declaration forms, and submits an entry request upon arrival (Figure 2a). This information is retained in a graph database (Figure 2b). Next, the application employs a radar map to assess visitor liability based on the self-declared data, incorporating their home address, places visited, and health condition (Figure 2c). The system subsequently approximates the venue's accessibility, taking into account the count of low-liability visitors inside, the potential contagion risk from nearby venues, and the venue's congestion level (Figure 2d). Ultimately, the system dynamically regulates visitor venue access or offers a suggested waiting period, determined by their liability and the venue's accessibility (Figure 2e). The subsequent sections provide a detailed explanation of the crucial steps of the framework.

## 4 Visitor liability profiling

In a public health emergency, data on visitor liability can be intricate and varied. Some applications declare they gather details such as visitor's age, email id, contact number, and zip code; the device's geographic location, unique identifiers, mobile internet protocol (IP) address, and operating system; along with the kinds of browsers utilized on the mobile device (17). However, such extensive data collection might infringe on the privacy of the visitors. Based on the hands-on experience in epidemic prevention and control (18), three key factors significantly influence visitor liability: *residency in high-risk areas, travel history to high-risk zones*, and *presence of specific symptoms*. These factors may correspond to the three necessary conditions of virus transmission mentioned in the infectious disease transmission model SEIR (19), viz., the source of infection, the route of transmission, and the susceptible populations. Consequently, we devise comprehensive voluntarily providing forms that solely encompass these three dimensions.

Moreover, it is not trivial to profile visitors' liability via these aspects. For example, some places judge whether to allow visitors to enter only based on their temperature. Since only one dimension is considered, the profiling is not comprehensive. Therefore, we build a radar map based on the information visitors provide and profile their liability by calculating its area. In the following, we introduce our solution to address these problems.

### 4.1 Voluntarily providing data collection

**Figure 3:**
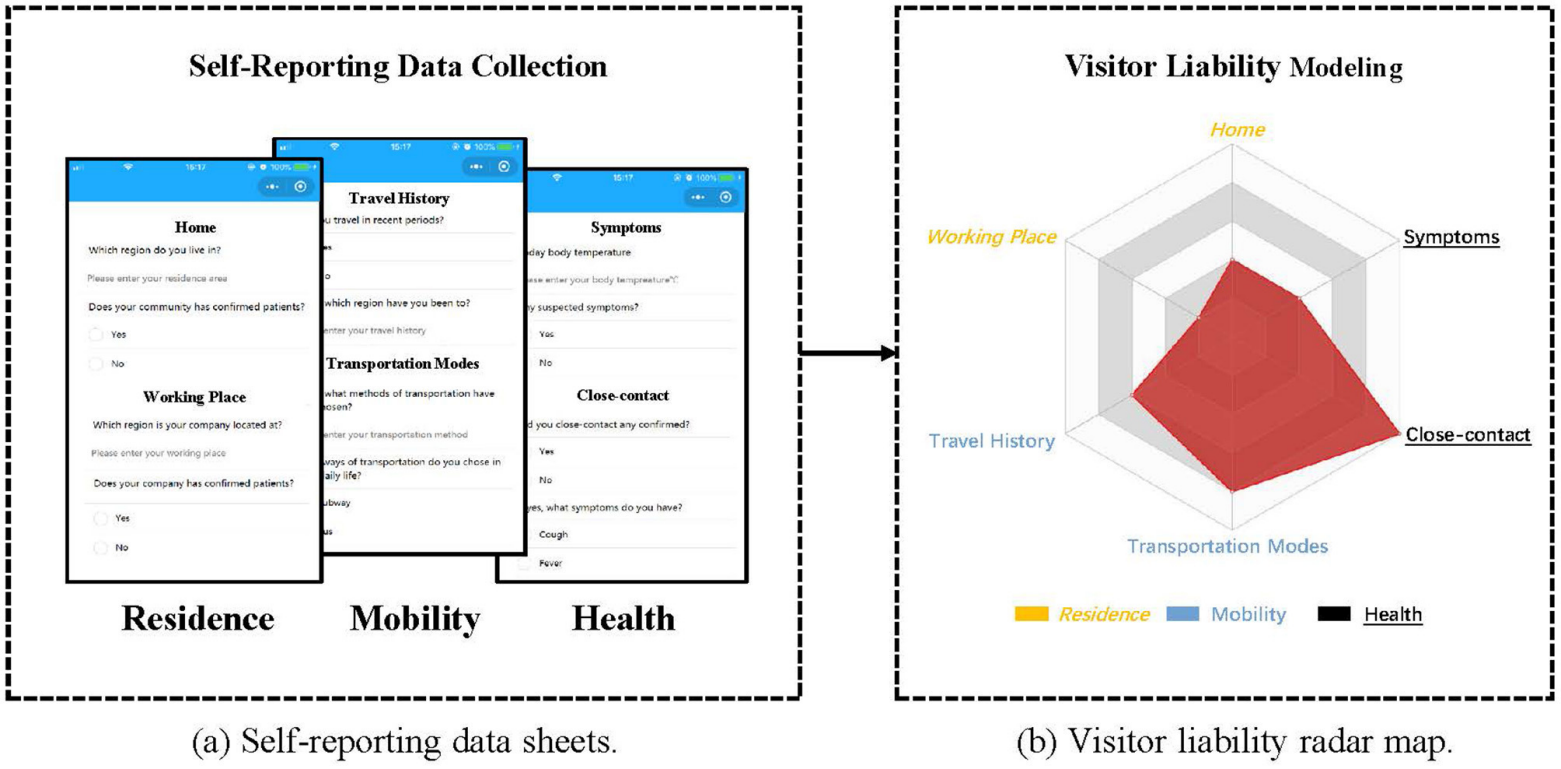
Overview of Visitor Liability Model. **(a)** The proposed voluntarily providing data sheets include 3 aspects, i.e., *Residence, Mobility* and *Health*. The answers are compared with our predefined rules to derive six corresponding scores. **(b)** The radar map is used to quantify visitor liability. The larger area corresponds to a higher visitor liability.

We create anonymous voluntarily providing forms for visitors to disclose merely three categories of information, as depicted in Figure 3a, thereby balancing the efficiency of form completion and the relevance of the provided data. Particularly, the *Residence* category encompasses the visitor's *Home* and *Workplace*, the *Mobility* category includes the visitor's *Travel Record* and *Means of Transport*, and the *Health* category involves the visitor's *Symptoms* and *Close Contact* instances.

In order to ensure the security of data, all collected data is encrypted during transmission and storage. We use advanced encryption algorithms, such as AES-256, to protect the data from unauthorized access. Additionally, all sensitive information is only used within the internal system, and external systems cannot directly access this data. Furthermore, we have implemented strict access control policies, allowing only authorized system administrators to access visitor data.

To quantify the details given by visitors, administrators of the venue can assign diverse scores to each category using adaptive scoring standards, which are adjusted based on varying phases of the epidemic situation. We provide a detailed explanation below.

Initially, we assess the vulnerability linked to the areas where visitors reside, including their *Home* and *Workplace*. This risk is rated based on regional risk grades, defined in five tiers, as updated by the government. Consequently, we allocate a liability rating ranging from one to five for each category. Next, we assess the potential risks associated with the visitor's *Travel Record* and *Means of Transport*. In particular, we designate a diminished liability score for congested public transport options such as metros and buses, while assigning a relatively elevated liability score to individual commuting methods like driving and walking. Finally, we assess elements related to the visitor's *Symptoms* and *Close Contact* instances, based on their severity and the changing pandemic conditions. For instance, when a visitor exhibits indications like elevated body temperature and respiratory distress, we designate a livability score that aligns with the guidelines for epidemic prevention and control (20).

In this study, we create a 6-dimensional vector *V* for each visitor, where each element of *V* corresponds to the visitor's rating for the respective item.

### 4.2 Visitor liability modeling

Using the previously mentioned factors, we develop a six-axis radar map and utilize it to evaluate visitors' liability by calculating its enclosed area. Illustrated in Figure 3b, the radar map comprises six endpoints including *Symptoms* representing *Health, Traveling History* representing *Mobility*, and *Home* representing *Residence*. These vertices are assigned scores that correspond to the respective factors' values. The radar map allows us to observe that a higher liability score for the six factors corresponds to a larger area, while even a slight variation in the axes' values can lead to a significant change in the area, making it an effective and sensitive method to finely assess visitors' liability. Consequently, by computing the area of the radar map formed by vector *V* and subsequently normalizing it, we obtain the visitor's liability score φ(*u*) using the approach proposed by Porter and Niksiar (21):


(1) $$\begin{array}{l} \varphi \left(u\right)=\frac{\sum_{i=0}^{a-1}\left(V_{i\%a}\cdot V_{\left(i+1\right)\%a}\right)}{\sum_{i=0}^{a-1}\left({\hat{V}}_{i\%a}\cdot {\hat{V}}_{\left(i+1\right)\%a}\right)} \end{array}$$

Given that *V* has *a* dimensions, where *V*_*i*_ and $\hat{V}i$ denote the value and upper limit of the *ith* component of *V*, respectively. If a visitor's liability score φ(*u*) is below the designated threshold θ_*liability*_, the visitor is categorized as a low-liability visitor. The value of θ_*liability*_ can be determined at the discretion of venue managers according to their specific requirements.

## 5 Venue availability monitoring

During a public health crisis, the availability of venues, which is considered a safety metric in this study, can be influenced by several factors, including the density of people within the venue, the proximity of neighboring venues, and the venue's inherent capacity. However, quantifying such information can be challenging. Hence, according to the infectious disease transmission model SEIR, we evaluate the venue availability from three factors as follows: First, visitors with low liability in the venue may have potential risks. Second, the transmission of visitors with low liability among venues may provide a potential risk for the virus to spread. Third, visitors are easily susceptible to crowded venues, because they are more likely to be in close contact, which leads to the risk of virus transmission increasing. In this section, we propose effective solutions to quantify these three factors to comprehensively estimate venue availability.

### 5.1 Venue member liability risk

The number of visitors with low liability in the venue is an important indicator to measure visitors' risk of the venue. Therefore, we calculate the visitor's risk *R*_1_ in the venue by using the following formula:


(2) $$\begin{array}{l} R_{1}=n_{l} \end{array}$$

where *n*_*l*_ represents the number of visitors with low liability. The larger *R*_1_ represents the lower venue availability.

### 5.2 Neighboring venue propagation risk

The venue's risk from the neighboring venue is related to the number of visitors with low liability that may be transferred from neighboring venues. We discover potential connections among venues based on the historical check-in records stored in the graph database. And we regard the number of potential low-liability visitors transferring from neighboring venues as the venue propagation risk *R*_2_, which can be calculated as follows:


(3) $$\begin{array}{l} R_{2}=\overset{b}{\underset{i=1}{\sum }}r_{i}\cdot \frac{n_{i}}{m_{i}} \end{array}$$

where *b* is the number of venues, *r*_*i*_ is the number of low-liability visitors in the *i*_*th*_ venue in current, *n*_*i*_ is the number of visitors transferred from the *i*_*th*_ venue to the current venue in history, and *m*_*i*_ is the total number of visitors leave from the *i*_*th*_ venue in history. The larger *R*_2_ represents the lower venue availability.

### 5.3 Venue crowdedness risk

The venue's crowdedness risk denotes the probability of the visitor's contact with another visitor in the venue. For example, a venue with a higher crowdedness risk is more likely to lead to more contact between visitors. We calculate the venue crowdedness risk *R*_3_ using the following equation:


(4) $$\begin{array}{l} R_{3}=\frac{N}{N_{c}} \end{array}$$

where *N* refers to the number of visitors in the venue, and *N*_*c*_ refers to the maximum capacity of the venue, which is set by the venue manager. The larger *R*_3_ represents the lower venue availability.

### 5.4 Venue availability calculation

Based on the above-mentioned three factors *R*_1_, *R*_2_ and *R*_3_, we evaluate the venue availability ω(*v*) by calculating the probability of a random visitor not getting infected in random contact with other visitors in the venue using the following equation:


(5) $$\begin{array}{l} \omega \left(v\right)={\left(1-\beta \right)}^{\left(R_{1}+R_{2}\right)\cdot R_{3}} \end{array}$$

In the Equation 5, β refers to the transmission rate when a people contact with a COVID-19 case, which is defined as *R*_0_/τ, where *R*_0_ is the basic reproduction number of virus (22), and τ represents the average number of person-to-person contact during the infectious period. Therefore, (1 − β) refers to the probability that a visitor will not get infected when they contact each visitor in the venue.

*R*_1_ refers to the number of low-liability visitors in the venue. *R*_2_ refers to the number of potential low-liability visitors transferring from neighboring venues to the current venue. Consequently, (*R*_1_ + *R*_2_) refers to the total number of low-liability visitors that the visitor may contact in the venue. The venue crowdedness risk *R*_3_ is used as a scaling factor to adjust the probability of a visitor getting in contact with a low-liability visitor. For example, *R*_3_ is large when the venue's visitor count exceeds the maximum capacity of the venue, which corresponds to more contact between visitors.

In conclusion, Equation 5 can represent the probability that a visitor will not get infected in the venue, taking into consideration the number of low-liability visitors in current and neighboring venues and the probability of contact among visitors.

## 6 Dynamic access control

During the public health crisis, to effectively avoid the influx of people into the venue during a specific period to reduce risks, we need to arrange a reasonable entry time for them individually. Intuitively, the venue managers can set thresholds of visitor liability and venue availability to control visitors' access to the venue. However, because different venues have different risk levels, venue managers have to set different thresholds for them, which requires human experience and research. Therefore, we propose to apply a queuing model based on M/M/s with non-preemptive priority to provide each visitor with an appropriate time to enter the venue to keep the number of visitors in the venue at a lower level, to lower the infection risks.

Visitor's priority is a concept in queuing theory (23). Visitors with lower priority usually need to wait a longer time than those with higher priority to receive service. For example, in the hospital queuing system, emergency patients usually have a higher priority to receive treatment first. Similarly, in our active access control strategy, we regard a visitor's liability score as their priority, i.e., visitors with higher liability scores have a higher priority to enter the venue, while visitors with lower liability scores need to wait for a specific period calculated by the system. The reasons are, on the one hand, we encourage visitors to fill in the voluntarily providing sheets completely to obtain higher liability (since unfilled items are assigned to zero scores). On the other hand, we also encourage visitors with low liability, such as those who traveled to high-risk regions recently or have suspected symptoms, to wait for the appropriate time to enter the venue, to reduce the potential risks that they may bring to the venue.

We first use visitor's liability φ(*u*) to denote visitor's priority *k* (a lower *k* corresponds to a higher priority) in the M/M/s queuing model as follows:


(6) $$\begin{array}{l} k=\varphi {\left(u\right)}^{-1} \end{array}$$

As a result, we use the venue's openness ω(*v*) to calculate the venue's available capacity *s* in the M/M/s queuing model as follows:


(7) $$\begin{array}{l} s=\omega \left(v\right)\cdot N_{c} \end{array}$$

where *N*_*c*_ represents the maximum capacity of the venue predefined by the venue manager. We assume that a venue with a lower availability leads to a lower available capacity.

Finally, based on the M/M/s queuing model, we calculate the average waiting time of the queuing system for a visitor with priority *k* (*k*=1,2,…,*m*) as *W*_*k*_:


(8) $$\begin{array}{l} W_{k}=\frac{{\left[s!\left(1-\rho \right)\left(s\mu \right)\sum_{i=0}^{s-1}{\left(s\rho \right)}^{\left(i-s\right)}/i!+s\mu \right]}^{-1}}{\left(1-\sigma_{k-1}\right)\left(1-\sigma_{k}\right)} \end{array}$$

where


(9) $$\begin{array}{l} \;\sigma_{r}=\{\begin{array}{cc} 1, & r=0\\ \sum_{i=1}^{r}\rho_{i}, & r>0 \end{array},\;\rho_{i}=\frac{\lambda_{i}}{s\mu },\;\rho =\frac{\lambda }{s\mu },\\ \;\lambda =\overset{m}{\underset{i=1}{\sum }}\lambda_{i},\;s.t.\;\rho_{i}<1 \end{array}$$

where λ_*i*_ represents the arrival rate of visitors with priority *i* which follows Poisson distribution, and λ represents the arrival rate of all visitors. λ_*i*_ can be estimated by (*n*_*i*_/*n*)λ, where *n*_*i*_ represents the number of visitors with priority *i*, and *n* represents the total number of visitors. The service rate μ means the number of visitors that the venue can serve per hour, which can be calculated by 1/*T*, where *T* represents the average time a visitor stays in the venue and follows Exponential distribution. In practice, we assume that the visitors arriving at the venue form a queue, and we assign the average waiting time *W*_*k*_ to the head of the queue. When the queue head enters the venue, the waiting time of other visitors is updated according to the new queue head.

## 7 Evaluation

In this segment, we assess the functionality and efficiency of the HealthPass implementation. To obtain comprehensive data for evaluation, we deploy an initial version of the system within the university campus and ensure its continuous operation starting from May 2020. Initially, a comprehensive statistical examination of the amassed dataset is performed to detail the implementation of the HealthPass system. Subsequently, we perform a set of simulation tests on the campus, using real-world data collected from the operational HealthPass system, to validate its efficiency. Lastly, we assess its usability and operational capabilities via a System Usability Scale (SUS) survey (24) and two case studies.

### 7.1 System deployment

We've created a web-based app for touch-free check-in, which provides visitors with convenience and imposes no additional burden, since check-ins can be completed using only the mobile phone's camera, without necessitating the download of any superfluous apps. On the server side, we've set up a relation graph database to archive the connections between visitors and venues. To safeguard the privacy and protection of the visitors, we allocate entirely random anonymous identifiers without following any particular pattern, and all data are encrypted.

**Figure 4:**
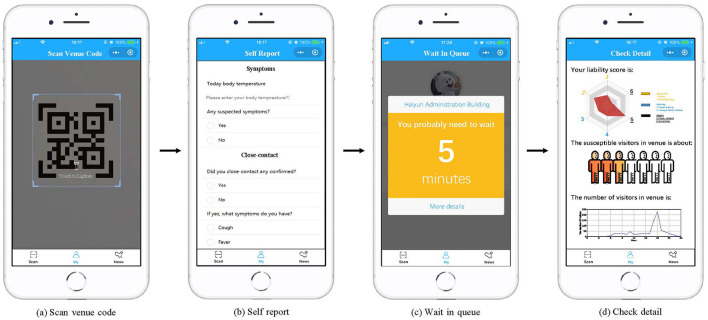
The user interface of the HealthPass system.

In practical implementation, the system initially creates a unique QR code carrying the venue's identifier for each location. Next, as depicted in Figure 4a, the visitor is prompted to complete voluntarily providing forms consisting of three sections, post scanning the venue's QR code with their mobile phone camera. Thus, the app is able to assess the visitor's liability accordingly (Figure 4b). In particular, visitors can fill in their reasons for entering the venue so that venue managers can better optimize corresponding services, which reduces the time that visitors spend in the venue. Third, the system will combine the visitor's risk assessment and the venue's capacity status to create a dynamic access control strategy. As shown in Figure 4c, the feedback page visitors receive contains their waiting times. Besides, three types of information are provided to help visitors have comprehensive knowledge about the three parts, as shown in Figure 4d. More specifically, the first part is a radar map displaying the visitor's liability, the second part is a chart showing the in-venue visitor liability risk, and the third part is a line chart showing the visitor count within the venue. To accurately track the visitor count currently in the venue, visitors need to scan the code again when they left the venue. As for people who are incapable of using technology, the on-site venue volunteers will help them record their voluntarily providing information to the system, and obtain waiting times for the visitors based on the system.

### 7.2 Statistical analysis

**Figure 5:**
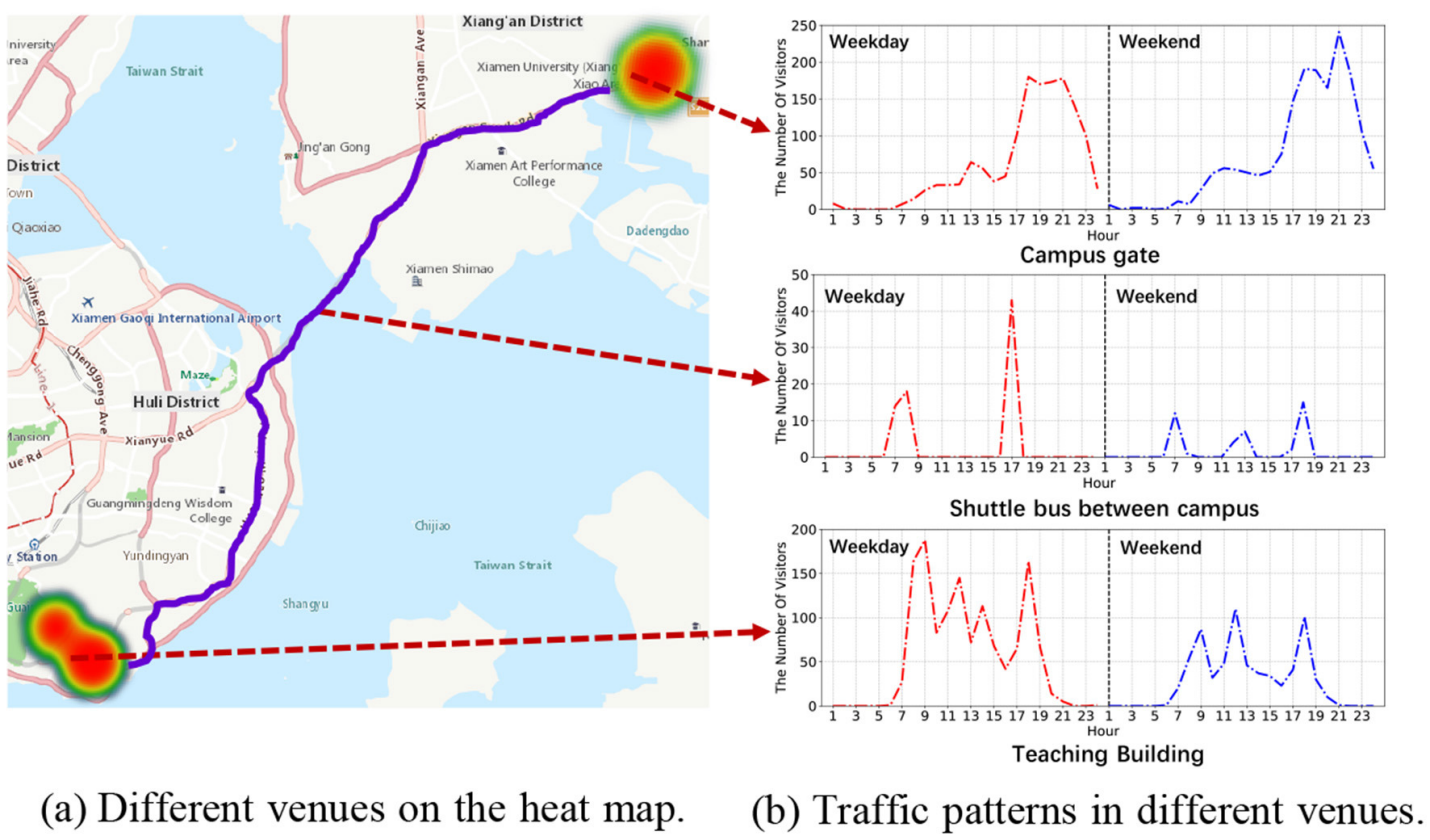
The recorded data on the utilization of the HealthPass system.

We have conducted an analytical study on the user behavior dataset that has been collected since May 2020 using the HealthPass system. This dataset comprises data from a total of 29,791 users and records 2,044,719 instances of visitors accessing 52 venues. Our statistical analysis indicates an average of 68.64 check-ins per user, while the most prolific user has contributed an impressive 3,663 check-ins. Also, the mean number of check-ins per venue amounts to 39,321.52, with the venue of highest popularity attracting 716,333 check-ins. Figure 5a illustrates the varying appeal of distinct venues. We delve into the analysis of the characteristics of regular weekdays and weekends at three typical venues: university entrances, shuttle transportation, and academic buildings.

The analysis, as presented in Figure 5b, highlights varied movement trends at distinct venues, offering valuable insights to effectively distribute resources for virus prevention.

### 7.3 Simulation experiment

**Figure 6:**
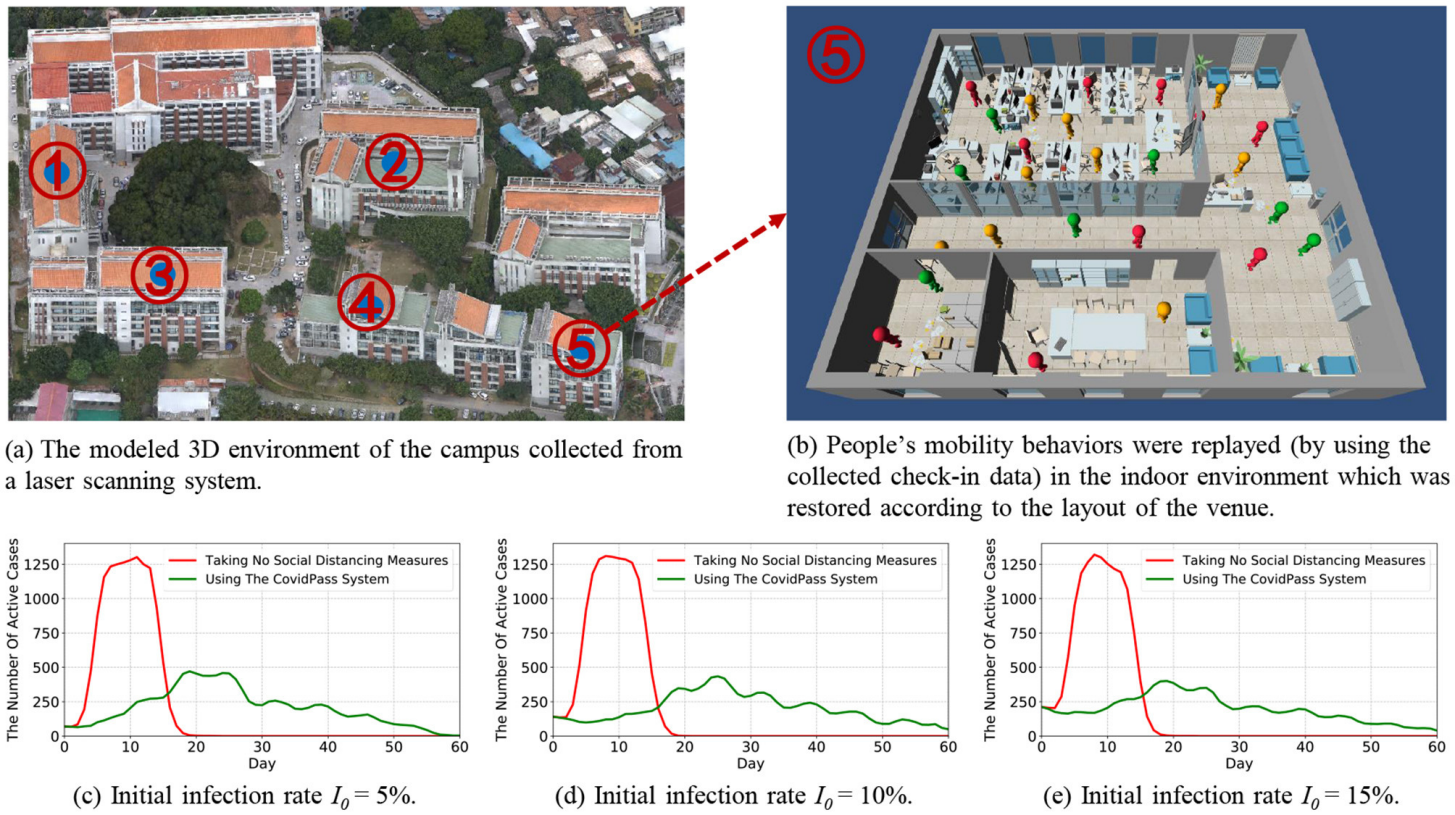
The overview of the simulation platform, and the comparison of the COVID-19 prevention effects of using the HealthPass system to that of taking no social distancing measures under different initial infection rate *I*_0_.

Since there were no COVID-19 infection cases reported on the university campus before and after the system deployment, we conduct a series of simulation experiments using the collected data to validate the performance of the HealthPass system. We take the Haiyun campus where the HealthPass system was deployed as an example for simulation experiments, involving 1,416 visitors and their 128,656 pairs of check-in and check-out records from 05/01/2020 to 07/01/2020 in five venues as shown in Figure 6a. This 3D model was generated using data collected by a laser scanning system, clearly indicating several important access points on campus. These venues represent critical areas in the implementation of the HealthPass system, which are used to accurately track the movement and contact paths of visitors. This provides realistic environmental data support for our simulation, making the experiments more closely aligned with real-world conditions and enhancing the validity of the results.

Based on the existing literature about epidemic prevention and control, one of the most important goals is to *flatten the curve* (25), which aims to mitigate the rapid spread of the epidemic, thereby lowering the peak number of individuals in need of care simultaneously, and preventing the healthcare system from being overwhelmed. Consequently, we proposed two metrics to access the effectiveness of the HealthPass system in *flattening the curve*, i.e., *reducing the peak number of active cases* and *extending the time until reaching the highest point (peak)*.

We build a simulation platform using real-world settings of the university campus and the collected check-in data to evaluate the effectiveness of the system under different circumstances. Specifically, we first model the 3D environment of the campus from a laser scanning system (as shown in Figure 6a), and restore the indoor environment according to the layout of the venue (as shown in Figure 6b).

Then, we set up the COVID-19-related parameters for the simulation platform using real-world information collected from the campus and the corresponding region. Specifically, (1) we set θ_*liability*_ as 0.8, which means that if the visitor's liability fall below 0.8, the visitor will be classified as a low-liability visitor; (2) We assess the risk of different regions based on an updated regional risk level provided by the government. (3) We assign the reproduction number of infection *R*_0_ (26) in the region of the campus with 2.24 (27), indicating that an infected case is likely to infect an average of 2.24 people during the period of infection; 4) We set an average of 50 contacts per infected case per day before they recover; 5) We set the infection time experienced by a patient to 21 days, which includes a 6-day asymptomatic period (28); 6) We set the initial infection rate *I*_0_ to 5%, 10%, and 15%, respectively, to observe the effects of our system in lowering the infection risks under different infection rates.

Finally, we imported the HealthPass system-collected check-in data from the campus into the simulation platform to replay people's mobility behaviors (as shown in Figure 6b). Specifically, (1) we randomly fill in the voluntarily providing sheets for each visitor to derive their liability scores, and we assume that the low-liability visitors are the infected case, so as to facilitate the simulation. Therefore, the initial infection rate *I*_0_ is the same as the initial ratio of low-liability visitors; (2) When the visitors check in at a venue, they are put in a waiting queue and given waiting times according to the access control strategy mentioned in Section 6; (3) Visitors move freely in the 3D environment of the venue, and they are infected according to the transmission rate β when they interact with an infected case. The infected visitors go through four stages in turn, i.e., exposed, asymptomatic, infectious, and recovered. As shown in Figure 6b, the green color represents the healthy visitors, including the uninfected visitors and the recovered visitors; the orange color represents the visitors who are in the asymptomatic stage without capable of infection; the red color represents the infectious visitors.

We compared the COVID-19 prevention effects of using the HealthPass system to that of taking no social distancing measures under different initial infection rates *I*_0_. Figures 6c–e show that, compared with taking no social distancing measures, the HealthPass system can effectively *flatten the curve* by reducing the peak number of active cases from 1302 by 63.82% to 471, and delay the time to reach the peak from 11 days to 19 days. When the initial infection rate is low (5%), as shown in Figure 6c, the HealthPass system is highly effective, quickly reducing the peak number of cases and extending the epidemic's duration. At a moderate rate of 10%, as shown in Figure 6d, controlling the outbreak becomes more complex, requiring more resources and stricter measures, although the system's effectiveness remains clear. When the initial rate reaches 15%, as shown in Figure 6e, the challenge intensifies with a sharp rise in cases and transmission speed; while the HealthPass system still helps, its role shifts more toward delaying the peak and easing the burden on healthcare resources. Overall, a health pass system would effectively reduce the risk of COVID-19 infections on campus and reduce the strain on healthcare resources.

### 7.4 Usability study

In this study, we use the SUS (System Usability Scale) to evaluate the HealthPass system, which is a set of usability evaluation scales widely used in academic and industrial fields. The SUS scale includes 5 positive descriptions and 5 negative descriptions (24). All the items on the voluntarily providing questionnaire use a five-point Likert scale from *Strongly* Disagree to *Strongly Agree*. The score of every single question is converted into 0-4, and the overall score is converted into a percentile result according to a calculation method: (∑(*positive description score* − 1) + ∑(5 − *negative description score*) * 2.5) (24). Concretely, the positively-worded statements are numbered 1, 3, 5, 7, and 9. In contrast, the statements numbered 2, 4, 6, 8 and 10 are negatively-worded. We divide the statements into two aspects (24): statements 4 and 10 measure the *Learnability* of the system and others can evaluate the *Usability*. Intuitively, the higher the overall score is, the better the user's evaluation of the system. In the same way, usability and learnability can also be measured by their average score respectively, which can be used to study the future improvement of the system.

We send out the survey through social networks and email to participants who have used the system during this period. At the same time, we also collect user's comments to better get their feedback. We have 74 participants in total, and half of them are female. The professions of the participants are various.

Table 2 shows the final results we have obtained for the questions designed in this survey. The higher the score is, the better the performance of the system. Based on the data collected, the overall SUS score of the HealthPass system is 84.6. At the same time, we also find that the *Usability* and *Learnability* scores are 2.57 and 3.61, respectively. According to the study of Bangor et al. (29), the HealthPass system generally achieves a *Good* rating, and it also achieved good performance in terms of *Usability* and *Learnability*.

**Table 2:** System Usability Scale Scores (24) (Higher scores imply better user experience. Note that the SUS scores for S1-S10, learnability and usability range from 0 to 4, while the overall SUS score ranged from 0 to 100.).

**SUS statements**	**Average score**
S1: I think that I would like to use this system frequently.	3.37
S2: I found the system unnecessarily complex.	2.55
S3: I thought the system was easy to use.	3.53
S4: I think that I would need the support of a technical person to be able to use this system.	3.62
S5: I found the various functions in this system were well integrated.	3.35
S6: I thought there was too much inconsistency in this system.	3.11
S7: I would imagine that most people would learn to use this system very quickly.	3.73
S8: I found the system very cumbersome to use.	3.35
S9: I felt very confident using the system.	3.62
S10: I need to learn a lot of things before I could get going with this system.	3.61
Learnability dimension (S4 and S7)	3.61
Usability dimension (other 8 statements)	2.57
Overall SUS score	84.59

In addition, through the specific analysis of the score of each sub-question, we gain more understanding of the evaluation. Among them, We can see the scores of S4, S7 and S10 are relatively high, and they did not encounter too many difficulties in the process, which proves that the HealthPass system offers a good user experience. However, we also find that the system scored only 2.55 in S2, which indicates that the system is still unnecessarily complex. We believe that the reason for this may be that the statements in the voluntarily providing sheet are cumbersome for users. Moreover, in real-life deployment, the pages are loaded slowly when there are too many visitors using the system at the same time.

### 7.5 Case study

**Figure 7:**
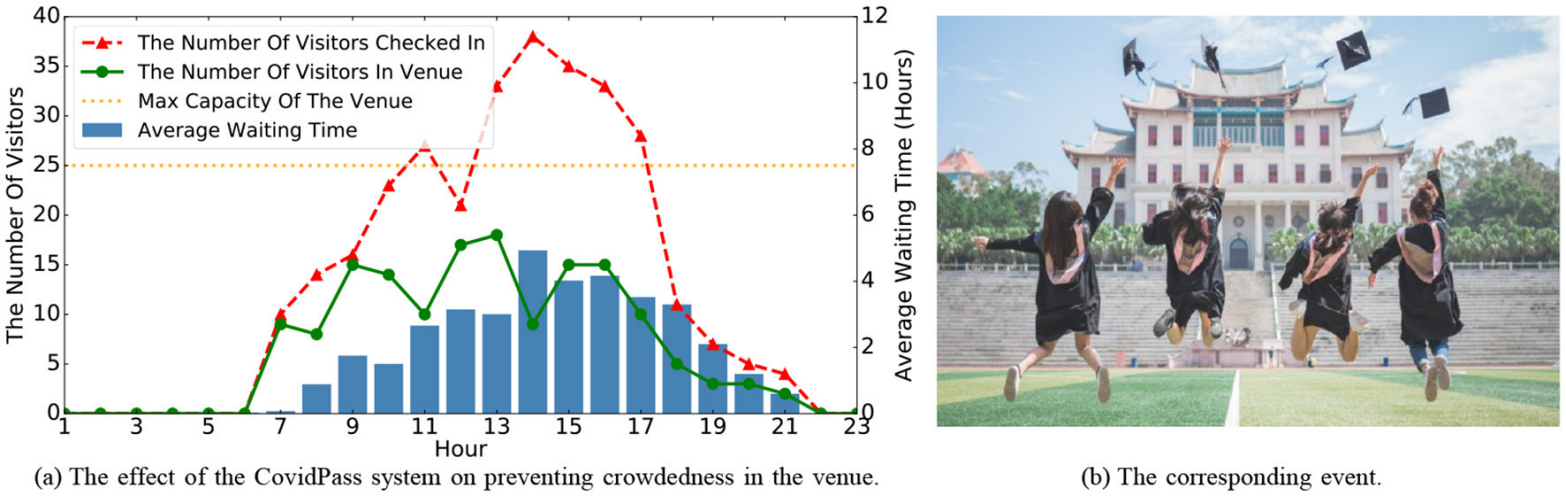
The effect of the HealthPass system on preventing crowdedness in an Office Building in the graduation season on 06/19/2020.

**Figure 8:**
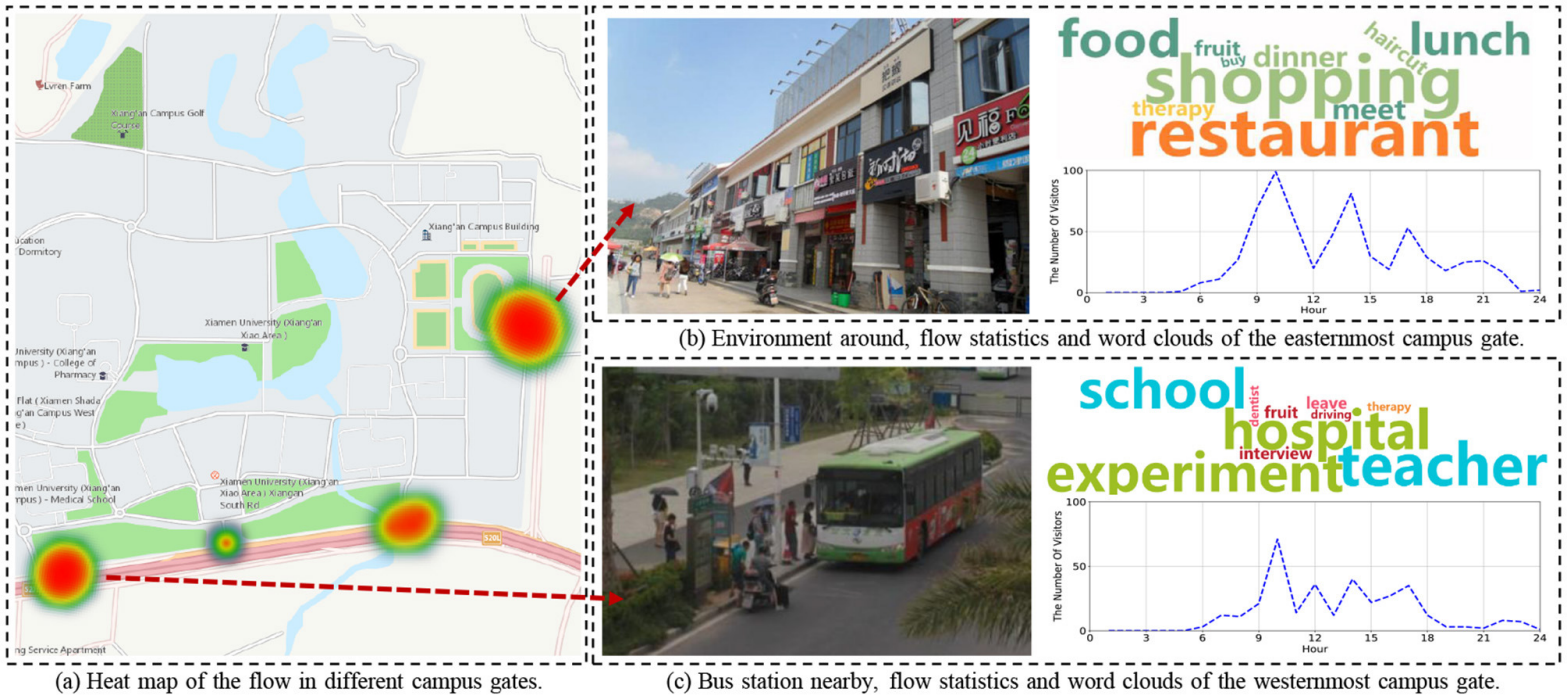
Word cloud of demands for leaving school.

Within this segment, we carry out two case studies to authenticate the performance of the HealthPass system. On the one hand, since special events happen sometimes and may cause crowdedness in the venue, it is necessary to validate the effectiveness of the HealthPass system in avoiding crowdedness in the venue. On the other hand, it is meaningful to analyze the reasons for entering the venue. In the subsequent examination, we provide two case studies of the collected data from the university campus. Figure 7 shows avoidance of a crowdedness event, and Figure 8 is the word cloud of users' demands on the campus.

#### 7.5.1 An office building during graduation season

In this case study, we take an Office Building as an example to validate the effectiveness of the HealthPass system in avoiding crowdedness and explaining the corresponding event. As shown in Figure 7b, we observe a potential crowding event in the venue. According to Figure 7a, the number of visitors entering the office building was significantly higher than usual between 11 am and 5 pm on 06/19/2020. Further investigation revealed that this was due to a large number of graduating students visiting the office building to handle graduation procedures, leading to a sharp increase in the number of visitors. To address this crowding situation during peak periods, the HealthPass system effectively maintained the number of visitors in the venue below the maximum capacity by extending the average waiting time. This approach helped visitors maintain proper social distancing within the venue, reduced the risk of contact, and prevented potential cluster infections, demonstrating the significant role of the HealthPass system in managing high-density crowd events.

#### 7.5.2 The new campus of a university

During the epidemic, it is suggested that people reduce outgoing to reduce the possibility of contacting outsiders for safety (30). However, due to imperfect campus facilities, people inevitably need to leave school to handle affairs. Therefore, it becomes very meaningful to analyze the reasons and flow of people leaving the school, because the infrastructure in the school can be improved through it. In this case study, we take the new campus of a university as an example, to count the reasons for going out and conduct a corresponding demand analysis. We receive 28,650 records and perform word frequency statistics on the reasons by adopting the word clouds.

The heat map in Figure 8a displays that most students go out from two school gates. First, as shown in Figure 8b, it summarizes the outgoing traffic of the easternmost gate at about 10 am, 1 pm, and 5 pm and the word cloud of the reasons mainly related to shopping, restaurant, and food. From the analysis of the surrounding environment there, it can be found that most of the service facilities provided are convenience stores, restaurants, and fruit shops. In response to this phenomenon, campus prevention and control strategies can be adapted to the pattern during these periods. Besides, the reasons why people go out through the westernmost gate are usually more inclined to study and work where we learn that there is a bus station nearby. Many people use this route to go to another campus of the university, which may explain the different phenomenon (as shown in Figure 8c) of this gate (the long distance between two campuses is shown in Figure 5b). In this regard, we consider providing shuttle buses for students to go out, and arrange shifts based on the peak traffic time shown, so as to reduce the possibility of unnecessary contact with outsiders, as well as to complete the purpose of epidemic control.

## 8 Discussion

### 8.1 Subjective initiative of voluntarily providing data

#### 8.1.1 Verify answers

While the assessment demonstrates the HealthPass system's ability to effectively schedule users' entry times and mitigate the potential for group infections, it is challenging to entirely prevent the chance of individuals attempting to manipulate the outcome to gain higher priority. Considering that users are only encouraged to report their data in the system, the information may be fake. We can verify the reliability of their answers by calculating the Cronbach coefficient of their reporting data, which can avoid fake answers more effectively. Also, it is believed that there is only a small part of the subjective behavior and does not represent others, but we will continue to improve the reliability and ease of use of the entire system.

#### 8.1.2 Incentives besides law enforcement

In this work, we consider user incentives from the following two perspectives. First, we encourage users to enrich their voluntarily providing sheets, the more accurate they fill in, the higher liability score they will get, and they will be more likely to enter the venues. Second, we obtain information about the anonymous ID of each user and their relations with different venues. Users can conveniently log in to the HealthPass app to review the locations they have visited. Furthermore, the HealthPass system proficiently identifies anonymous users potentially at risk of exposure by scrutinizing the infection sequence derived from the accumulated venue attendance data. Such users will be promptly alerted through messages, enabling them to promptly adopt appropriate precautions, such as going to the hospital for nucleic acid testing.

### 8.2 Ethical consideration

#### 8.2.1 Information collection

The data we gather is solely employed for generating visitors' liability scores within the system, ensuring that the particular input details remain confined and are not uploaded. Furthermore, we meticulously crafted the voluntarily providing forms to guarantee visitors' convenient completion under the premise of 1) minimal questions, 2) straightforward response methods, and 3) minimal exposure to private information.

#### 8.2.2 Information storage

As visitors access venues utilizing anonymous identifiers, the stored check-in data remains devoid of any actual personal identification.

#### 8.2.3 Operation body

The university's COVID-19 control and prevention committee granted official endorsement to the HealthPass system, which was subsequently implemented and managed by the university's information center.

### 8.3 Usability in non-COVID times

The HealthPass system can be useful in dynamically controlling crowds based on available resources at a particular place. For example, the system can be deployed in hospitals to schedule patients efficiently according to their conditions and the availability of medical resources (e.g., the number of doctors). The system can also be used in large-scale social activities which need to control the human flow to ensure safety. For example, the serious stampede in the 2014 Shanghai New Year celebration (31) might be prevented if the HealthPass system was deployed for human flow control.

### 8.4 Limitations and future improvements

The limitation of the HealthPass system lies in the lack of experimental data for direct comparison with other non-contact registration systems. This constraint is mainly due to our emphasis on user privacy, ensuring that all visitor data is anonymized, which prevents us from obtaining data from other systems for comparison. Additionally, data collection limitations during the pandemic have made acquiring experimental data from other systems challenging. In the future, we will optimize the HealthPass system and expand its application scenarios, while also conducting comparative analyses with traditional paper registration systems and other electronic registration systems to further validate its effectiveness and practicality, enhancing its potential in public health crises.

### 8.5 Further experiment

To investigate the effectiveness of machine learning-based approaches in COVID prevention and venue access control, we also conducted a pilot experiment to predict the pressure of medical institutions in a city. We build a spatiotemporal prediction model which uses a recurrent neural network (RNN) to capture the time dependencies and a graph neural network (GNN) to model the spatial correlations.

## 9 Conclusion

In our study, we present the HealthPass, an automated check-in solution that integrates anonymous visitors' voluntarily provided to establish an active venue access strategy informed by both the visitor's risk assessment and the venue's capacity status. Initiating, we evaluate the visitor's level of responsibility derived from the information they furnish through radar map analysis. Next, we gauge the accessibility of venues by quantifying the potential for low-liability members in the venue, the transmission risk from neighboring venues, and the potential for crowdedness against social distancing. Third, a queuing model is utilized to control visitor access in an adaptable manner, considering the extent of the risk posed by visitors and the capacity status of the venue. Utilizing the real-world implementation, we carry out a series of simulation experiments and case studies to confirm the effectiveness of the HealthPass system in lowering infection risks.

Moving forward, we plan to enhance this study by addressing the following areas. Initially, we aim to incorporate additional data analysis methods to achieve a more comprehensive quantitative understanding of users' check-in behaviors in terms of common patterns and abnormal patterns. Second, we plan to go deep into studying the cognitive psychology of users to evaluate their behavior patterns when interacting with the HealthPass system. Third, we intend to broaden our scope by incorporating additional factors, thus enhancing the adaptability of the access control approach.

## Data Availability

The original contributions presented in the study are included in the article/supplementary material, further inquiries can be directed to the corresponding authors.

## Footnotes

^1^ https://coronavirus.jhu.edu
